# Organic-Free Synthesis of Finned Mordenite Zeolite

**DOI:** 10.3390/nano12152623

**Published:** 2022-07-29

**Authors:** Rafael C. Lima, Christian W. Lopes, Jhonny Villarroel-Rocha, Lindiane Bieseki, Karim Sapag, Sibele B. C. Pergher

**Affiliations:** 1 Laboratory of Molecular Sieves, Institute of Chemistry, Federal University of Rio Grande do Norte, Natal 59078-970, Brazil; rafael_ibc@yahoo.com.br (R.C.L.); lindiane.bieseki@gmail.com (L.B.); 2 Laboratory of Reactivity and Catalysis, Institute of Chemistry, Federal University of Rio Grande do Sul, Porto Alegre 91501-970, Brazil; chriswittee@gmail.com; 3 Laboratory of Porous Solids, Institute of Physics, University of San Luis, San Luis 5700, Argentina; jhoviro@gmail.com (J.V.-R.); sapag@unsl.edu.ar (K.S.)

**Keywords:** zeolite, mordenite, organic-free synthesis, morphology, finned particles

## Abstract

Mordenite is a well-known zeolite widely used for industrial processes. However, its pore architecture can be inconvenient due to diffusional issues. A study of the synthesis parameters from an organic-free dense gel was carried out to control the crystal morphology, which resulted in finned mordenite zeolite particles. The obtained materials were characterized by XRD, FTIR, ^29^Si and ^27^Al MAS-NMR, elemental analysis, nitrogen physisorption, SEM, and TEM. We found that careful manipulation of the hydrothermal parameters directly affected the sizes and morphologies of the crystallites and particles, as well as the textural properties of the final products. Additionally, it was found that mordenite could exhibit a fin morphology with additional mesoporosity, which is a promising means to reduce the diffusional problems of one-dimensional-channel zeolites.

## 1. Introduction

Mordenite is a well-known, high-silica aluminosilicate zeolite present in natural deposits, but it can also be synthesized by several routes [1]. Its structure is represented by the idealized chemical composition of Na_8_Al_8_Si_40_O_96_·nH_2_O. Mordenite’s pore system consists of two 8MR (membered-ring) channels in the *b*- and *c*-axes and one 12MR channel, known as the principal channel, in the *c*-axis, contributing to the performance of this zeolite in catalysis. All the channels are one-dimensional, although the side-pocket and 8MR channels intersect with the principal channels [2].

Mordenite’s high thermal stability and acid resistance, as well as the presence of active sites have made it one of the most-used zeolites in industry. It is a member of the “big five”, i.e., the most commonly used zeolites in catalysis; in processes such as cracking, alkylation, dewaxing, and reforming [3]; and in separation and adsorption processes. Additionally, mordenite can be employed in processes outside the catalysis field, and statistics show [1] that, despite this zeolite having been studied for decades, there are unexplored aspects to be further studied.

Zeolite size and morphology manipulations have been an area of great research interest. The control of these parameters provides a way to make these materials more effective in several applications [4,5] since zeolite crystal and particle size modifications affect their performances. As catalysts, larger crystals can be more selective [6], while smaller crystal sizes confer greater deactivation resistance [7]. Variations in morphology may also influence the catalytic results of zeolites [8].

In the case of mordenite, the crystals tend to grow according to the unidirectional 12MR channel [5], which is the principal channel that allows the transport of molecules (although the 8MR channel plays a vital role in the confinement effects of these molecules) [2]. This preferential direction of growth can be inconvenient for the application of mordenite as a catalyst because of the increased chance of coke formation in these pores [9]. To overcome this issue, mesopore openings and hierarchical assemblies of zeolites have been commonly applied in the literature. Nevertheless, some studies have suggested ways to control mordenite crystal and particle sizes, but most have included the use of organic compounds or other additions to traditional hydrothermal synthesis procedures [1].

Therefore, studies aiming to better understand mordenite phase formation and to gain some control over the size and morphology of this material have attracted interest [10,11,12]. However, obtaining a nanosized or nanocrystalline version of this zeolite has been challenging due to the apparent necessity of alkali cations in the synthesis gel [13].

Recently, a new concept for zeolite particles was proposed in the literature, related to the synthesis of nondense zeolite particles composed of aggregated, nanosized crystallites [14]. These particles have the potential for circumventing the diffusional difficulties in zeolites with long channels.

Thus, this work aims to clarify how the formation of the mordenite phase occurs from an organic-free amorphous gel and how the synthesis parameters affect the sizes and the morphologies of the formed particles. The structural, textural, and elemental analysis results obtained in the present study show that careful adjustment of synthesis parameters and hydrothermal treatment conditions can modify the characteristics of the particles obtained and can produce finned particles of this zeolite in an organic-free reactional system without any competing phases. The influences of the heat treatment conditions, temperature, time, and gel dilution were evaluated, emphasizing the ultimate texture, size, and morphology of mordenite zeolites.

## 2. Materials and Methods

### 2.1. Synthesis Procedure and Reference Synthesis

For this study, the gel molar composition was set to 4.3 Na_2_O: 1 Al_2_O_3_: 30 SiO_2_: 485 H_2_O. The gel was obtained in a similar process as found in IZA Verified Zeolite Syntheses [15], as follows. An alkaline solution was prepared using NaOH (≥98%, Sigma Aldrich, St. Louis, MO, USA) and NaAlO_2_ (56% Al_2_O_3_, 45% Na_2_O, Riedel de-Häen, Seelze, Germany), which were added to double-deionized water; then, the resulting solution was diluted to the appropriate water concentration. Subsequently, a fumed silica source (Degussa, Munich, Germany, Aerosil^®^ 200) was added to the solution, and the mixture was mechanically stirred for 25 min. The resulting gel was transferred to a Teflon-lined stainless steel autoclave and subjected to hydrothermal treatment under different conditions (time and temperature).

### 2.2. Influence of Hydrothermal Treatment Conditions

Two hydrothermal treatment conditions were studied: static and tumbling at 32 rpm. In these studies, the crystallization temperature was fixed at 170 °C. The prepared gel was distributed in different autoclaves and removed from the oven after 6 h, 10 h, and 24 h of thermal treatment.

### 2.3. Influence of Temperature and Time of Synthesis

The synthesis kinetics of the mordenite zeolites were then analyzed at 170 °C and 150 °C with agitation at 32 rpm. Based on the results of the crystallization process, the samples were removed from the oven at time intervals of 6 h, 10 h, and 24 h after starting the thermal treatment.

### 2.4. Influence of Gel Dilution

In this part of the study, the as-prepared gel was diluted in a water equivalent to 50 wt% gel. The mixture was stirred for 25 min until complete homogenization and then subjected to heat treatment at 170 °C and 150 °C in an oven with programmed tumbling at 32 rpm. Different times (10–34 h) were studied; thus, the crystallization process was monitored as a function of time.

### 2.5. Influence of Sodium Content

The influence of the concentration of alkali cations in the synthesis was evaluated by modifying the molar composition of the following synthesis gel: x Na_2_O: 1 Al_2_O_3_: 30 SiO_2_: 485 H_2_O (where x = 2 (Si/Na = 7.5) or the same Si/Na molar ratio of the IZA recipe for mordenite at 6 (Si/Na = 2.5)).

### 2.6. Characterization

The crystalline structure of the solids was evaluated using X-ray diffraction with a Bruker D2 Phaser^®^ X-ray diffractometer using CuKα radiation with a Ni filter, with a current of 10 mA c and a voltage of 30 kV over the 5–50° 2θ range and with rotation at 15.0 rpm. The analysis was performed with a divergent slit of 0.6 mm and an anti-air scattering of 1.0 mm. Scanning was performed stepwise with 0.01° increments and an accumulation time of 0.1 s. The lower and upper discrimination values were 0.11 V and 0.25 V, respectively. Signal collection was performed with a LYNXEYE^TM^ (Bruker, Billerica, MA, USA) detector (192 channels).

Crystallization curves were elaborated from the normalized sum of the area integrals of the five most intense reflections (at approximately 2θ = 9.7°, 19.6°, 22.4°, 25.8°, and 26.4°), which were chosen for the characterization of the mordenite phase in each sample. [10]

FTIR spectra were obtained using a Perkin Elmer (Waltham, MA, USA) Spectrum 65 FT-IR instrument employing a KBr (Merk-Uvasol^®^, spectroscopic grade) pellet technique at a 1:1000 ratio of zeolite KBr and a wavenumber analysis range of 1600–400 cm^−1^. One aliquot of the as-prepared hydrogel was quickly frozen in liquid N_2_ and lyophilized for 24 h; then, this sample was analyzed using FTIR.

Solid-state NMR spectra were recorded at room temperature with a Bruker AV 400 spectrometer. ^29^Si MAS-NMR spectra were recorded with a spinning rate of 5 kHz at 79.459 MHz and a 55° pulse length of 3.5 μs with a repetition time of 180 s. ^27^Al MAS-NMR spectra were recorded with a spinning rate of 10 kHz and a 9° pulse length of 0.5 μs with a 1 s repetition time. The ^29^Si and ^27^Al chemical shifts were measured relative to tetramethylsilane and [Al(H_2_O)_6_]^3+^, respectively.

The chemical analysis was performed using inductively coupled plasma optical emission spectrometers (ICP-OES) with a Varian 715-ES instrument (Palo Alto, CA, USA) after the dissolution of the solid samples in a HNO_3_/HCl/HF aqueous solution. The analysis of the samples in the liquid phase was obtained with a Thermo Fisher iCAP 6300 Duo instrument (Waltham, MA, USA) using USEPA 6010C methodology.

Nitrogen adsorption and desorption isotherms at −196 °C were performed with a Micromeritics ASAP 2020 adsorption analyzer (Norcross, GA, USA). Before analysis, the samples were outgassed at 150 °C for 12 h. From the nitrogen adsorption data, the specific surface area (*S*_BET_) was evaluated using the Brunauer, Emmett, and Teller (BET) method [16], taking into account the IUPAC recommendations [17]; the micropore volume (*V*_μP_) and the external specific surface area (*S*_ext_) were calculated using the α_S_-plot method [18] with macroporous silica LiChrospher Si-1000 as the reference material, [19] and the total pore volume (*V*_TP_) was estimated by the Gurvich rule at 0.985 of *p*/*p*^0^ [20].

The SEM images shown in this work were obtained either with a Zeiss LEO 1450VP^®^ microscope operating at 18 kV or with a Zeiss Ultra-55^®^ (Oberkochen, Germany) operating at 1 kV, and the TEM micrographs were obtained with a JEOL JEM-2100F instrument (Tokyo, Japan).

## 3. Results and Discussion

### 3.1. Influence of Thermal Treatment Conditions: Agitation and Time

**Figure 1:**
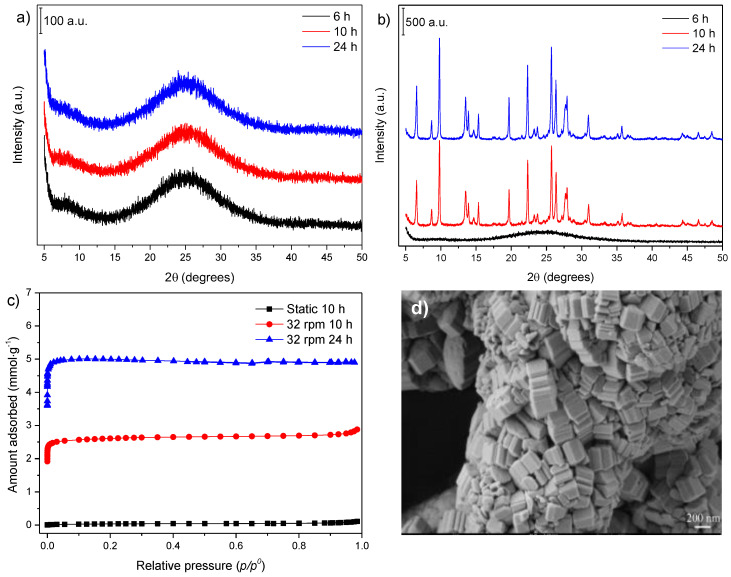
XRD patterns of samples synthesized under (**a**) static and (**b**) 32 rpm agitation conditions; (**c**) N_2_ adsorption–desorption isotherms, and (**d**) SEM image of the sample obtained after 24 h at 32 rpm.

The X-ray diffractograms presented in Figure 1a,b correspond to the samples obtained for the two heat treatment conditions studied: under static and under agitation (or by tumbling the autoclave at 32 rpm), respectively. The crystallization temperature was fixed at 170 °C.

For the static conditions (Figure 1a), the diffractograms did not present any reflection, suggesting the existence of an amorphous phase only. The synthesis reported by Kim and Ahn [21] from the IZA compendium generated a gel with a molar composition of 6 Na_2_O: 1 Al_2_O_3_: 30 SiO_2_: 780 H_2_O and used similar reagents to those employed in the present study. The use of a different silicon source can be listed as one of the factors for the nonformation of mordenite in static conditions since zeolite synthesis depends on the nature of this component [22]. Another possibility is the effect of the sodium content, which is essential for the formation of the MOR (mnemonic code for mordenite topology) phase [13] and was present in a greater concentration (4.3 in this work vs. 6.0 in the IZA synthesis).

When the synthesis was performed under agitation at 32 rpm (Figure 1b), which was adopted as a reference procedure in this work, 10 h of heat treatment resulted in the formation of the mordenite phase with no additional reflections from the other phases.

The N_2_ adsorption–desorption isotherm (Figure 1c) for the mordenite sample synthesized under static conditions (Entry 5 from Table 1) presented a negligible amount of N_2_ adsorbed, as expected for amorphous phases. In the case of the synthesis performed with agitation at 32 rpm, the isotherms of the 10 h and 24 h samples (Figure 1c) showed increased amounts of adsorbed N_2_ (the corresponding textural properties are presented in Entries 3 and 4, respectively). The sample obtained at 24 h displayed textural properties according to the bibliography for mordenite [23]. However, smaller values were obtained for the sample synthesized during 10 h of thermal treatment, indicating that the formation of the mordenite phase was not completed in these synthesis conditions, which agreed with the XRD data.

The SEM image of the sample obtained with agitation for 24 h of thermal treatment (Figure 1d) indicated the formation of crystallite aggregates whose morphologies tended towards the characteristic shape of mordenite zeolite crystals [24].

### 3.2. Influence of Temperature and Time of Synthesis

**Figure 2:**
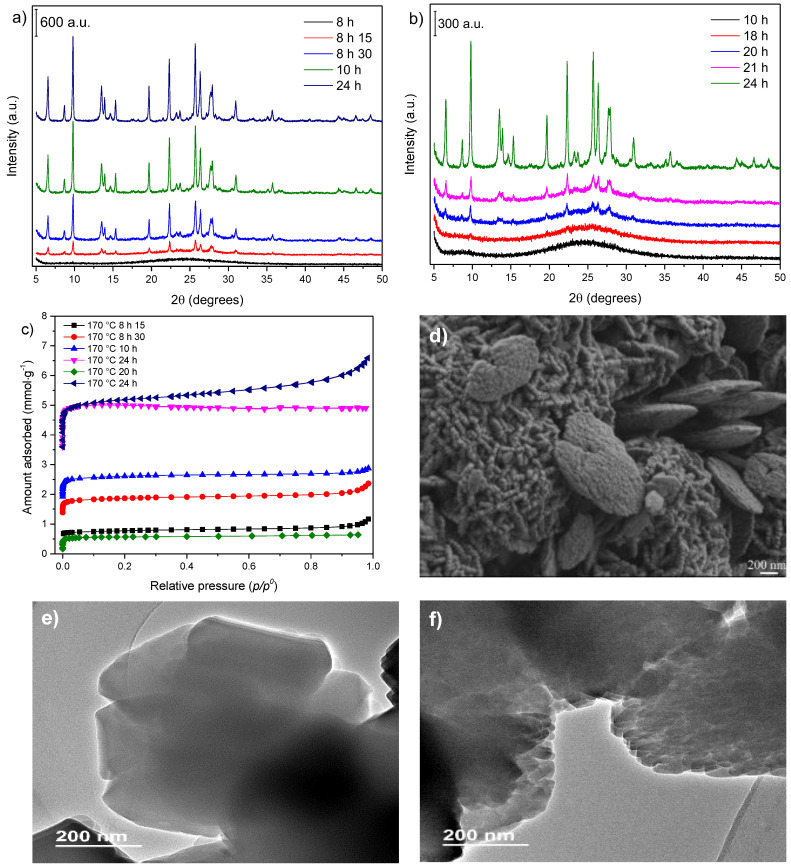
XRD patterns of samples obtained at (**a**) 170 °C and (**b**) 150 °C; (**c**) N_2_ adsorption–desorption isotherms; (**d**) SEM micrograph of the sample prepared at 150 °C for 24 h; (**e**) TEM micrographs of the samples synthesized at 170 °C for 24 h and (**f**) at 150 °C for 24 h.

Two synthesis temperatures (170 and 150 °C) were selected to study the temperature effect on the hydrogel system (Figure 2). The progressive transition of the amorphous primary gel to the mordenite phase was followed by characterization techniques at the two temperatures used in the present work and under 32 rpm agitation.

The XRD patterns for the samples obtained at 170 °C (Figure 2a) showed that there was a time interval (of about 15 min) between what could be called the induction period, as indicated by the characteristic amorphous phase (the 8 h sample) and the occurrence of all the typical reflections of the mordenite phase (the 8 h 150 °C sample). Thus, for the conditions imposed, as soon as the breakdown of the energy barrier between the precursor species and crystal formation occurred, crystal growth proceeded rapidly [25].

**Figure A1:**
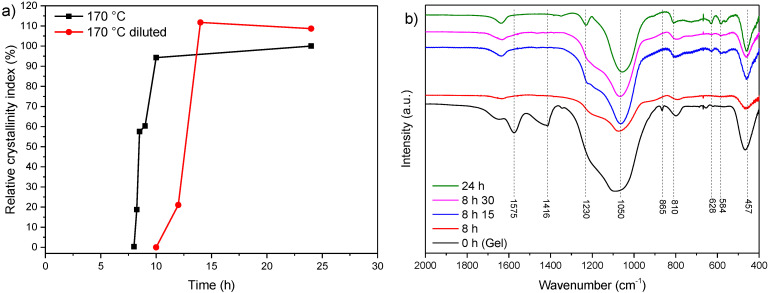
(**a**) Comparison of the crystal growth curves at 170 °C and 150 °C; (**b**) IR spectra accompanying the synthesis at 170 °C.

The effect of temperature on the crystallization rate of mordenite is illustrated by its crystallization curves (Figure A1a in Appendix A). The curve representing the synthesis at 170 °C indicated fairly abrupt crystallization between 8 and 9 h of thermal treatment, while the curve at 150 °C showed a more gradual progression in the growth of the crystals, with the first signs of crystals detected after 18 h of treatment.

The crystal growth curve found in Kim and Ahn’s work [21] showed a somewhat shorter induction time, although the higher relative crystallinity percentage was also reached at approximately 18 h; this indicates that the crystallization was slightly less sudden. The crystallization kinetic curves obtained by Zhang et al. [26] and Zhang et al. [12] can also be compared with those in this work. In the first case, using fumed silica and 164 °C thermal treatment, the time to reach 100% relative crystallinity was approximately 70 h under conditions designated as dynamic. In the second work, the synthesis was performed using silica gel and under 170 °C, which required about 120 h for the complete crystallization of mordenite.

The reduced temperature slowed the crystallization process for the synthesis carried out at 150 °C (Figure 2b), indicating a more extended induction period. Moreover, the crystal growth and the consequent consumption of the amorphous phase material were more gradual, contributing to the increase in smaller particles.

Concerning the N_2_ adsorption–desorption isotherms (Figure 2c), a gradual increase in microporosity was observed with the increase in heating time. The specific surface area (Table 1) enlarged considerably within the sample in which the crystal growth started (Entry 1) and for the samples of the following two hours (Entries 2–3). The isotherms of the samples prepared at 170 °C showed an expected progressive increase in the amount of N_2_ adsorbed with the increase in crystallization time. The isotherms of the samples synthesized at 150 °C (Figure 2c and Entries 6–7 for textural parameters) exhibited similar behavior, but more gradual, to that of the samples at 170 °C. Furthermore, the isotherm of the sample prepared for 24 h at 150 °C (Entry 7) showed a small increase in the amounts of N_2_ adsorbed at higher relative pressures, indicating mesoporosity in the solid.

The FTIR spectra (Figure A1b) showed that, even before the heat treatment, the hydrogel exhibited several bands similar to those found in a well-crystallized zeolite sample. The band around 1050 cm^−1^, assigned to the external asymmetrical stretch between TO4 units, was the most intense band and was broadened in the completely amorphous system compared to the system with growing crystals. Shukla and Pandya [27] also reported this feature for the FTIR spectrum of amorphous silica.

The band at the 800 cm^−1^ range referred to symmetrical stretching (←OTO→) vibrations between tetrahedra, which suggests that particles in the as-prepared gel had interconnected tetrahedra that were equivalent to those in their crystalline peers in both number and bond type. Finally, the band at 460 cm^−1^ indicated the presence of double rings, which are the D5R characteristic of MOR topology [28].

After 8 h of heat treatment, the band intensities decreased, indicating that some of the initial solid passed into the solution or that some bonds were broken and reformed to form the zeolite particles. The most significant change in the spectra was the disappearance of the bands at 1575 cm^−1^ and 1400 cm^−1^, making it clear that they referred to bonds that had no part in the zeolite structure, disappearing completely when the crystallization process began.

The next vibration block was related to the zeolite structure. A shoulder began to form near 1200 cm^−1^ due to internal asymmetric stretch vibrations in the tetrahedral units. This band was more strongly associated with oxygen atoms and became progressively more defined as crystallization occurred. The band at 1065 cm^−1^ underwent tiny shifts as the zeolite was formed, which could be related to the polymerization of the tetrahedra and the aluminum transport of the solution phase to the hydrogel [29,30].

The bands in the 900–500 cm^−1^ region, where most of the vibrations of intertetrahedral bonds lie, showed the progressive appearance of small bands characteristic of zeolitic structures over time. The band at 800 cm^−1^ was susceptible to structural modifications, and its shape changed as the gel passed entirely from an amorphous to a crystalline phase [28,31].

Moreover, the low and wide band at 725 cm^−1^ could be assigned to the deformation vibration of the S4R rings and other vibrational modes [28]. These rings may be those that bind to five-membered rings to form mordenite chains or those that form when the MOR structure is observed as interconnected lamellas. This band may also be the result of the overlapping of narrower bands.

The bands in the 580–550 cm^−1^ region could be linked to the structural order of zeolite or vibrations in the 8MR channel [32]. Most likely, these rings must be the windows of the 8MR channels and the side pockets of the MOR structure, despite the pore-opening vibrations being shown in the far-infrared region [15].

The weak band at 549 cm^−1^ may be associated with rotational vibrations or may be attributed to vibrations of the D5R rings. In addition, the bands at 628 cm^−1^ and at 457 cm^−1^ were attributed to the deformation of these rings [28,32,33,34], which only undergo variations in intensity as the amorphous silicate gives way to the mordenite phase.

The temperature effect on the morphologies and sizes of particles and crystallites can be observed by comparing the SEM images of the sample synthesized at 150 °C (Figure 2d) with the corresponding sample prepared at 170 °C (Figure 1d). At 150 °C, the crystallites aggregated into two shapes of particles, and some of them showed a larger length but with a small thickness. Moreover, the crystallites did not maintain the same behavior as those for the sample at 170 °C.

**Figure A2:**
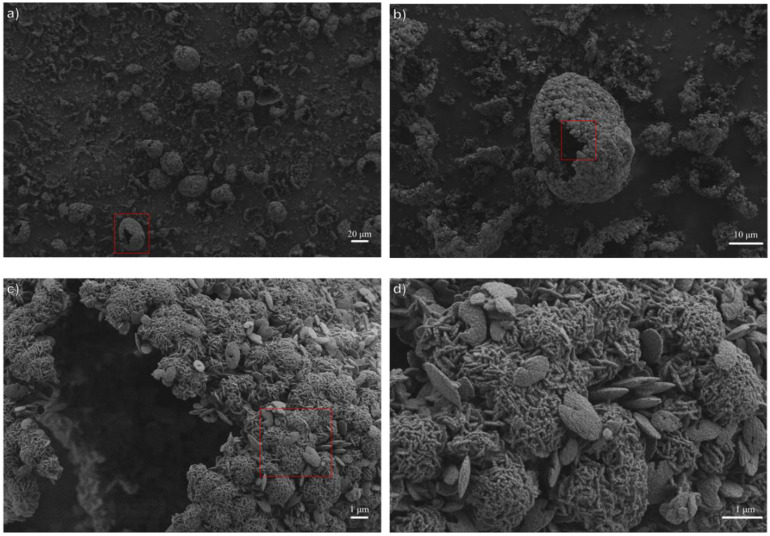
(**a**–**d**) SEM images showing the clusters of the sample obtained at 150 °C and 24 h at different magnifications. Red squares stand for the higher magnification of the following image.

In addition, the reduction in the crystallite size was evident. For the sample synthesized at 170 °C, the sizes varied from 270–100 nm, and for the solids prepared at 150 °C, the size range was 80–30 nm. A comparison of the TEM images (Figure 2e,f) confirmed these values, and it was seen that the shape of the crystallites obtained at 150 °C was significantly different from that of the reference sample prepared at the same time (Figure 2f). Interestingly, the aggregates formed by these nanocrystallites tended to agglomerate in a hollow, sphere-like morphology (Figure A2a–d). Therefore, the TEM images revealed the character of the finned particles of the sample at 150 °C, while at 170 °C, the particles were denser.

**Figure 3:**
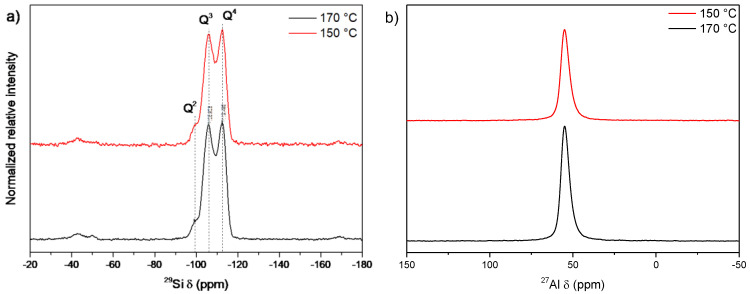
Solid-state NMR spectra of (**a**) ^29^Si and (**b**) ^27^Al in the samples synthesized at 170 °C and 150 °C for 24 h.

The samples synthesized during 24 h of thermal treatment at 170 and 150 °C were analyzed using solid-state NMR (Figure 3). The results indicated no considerable structural composition variations between the samples prepared at the two evaluated temperatures.

The ^29^Si NMR spectra of the samples prepared at both 170 °C and 150 °C after 24 h (Figure 3a) showed three signals. The first, centered at −112 ppm, corresponded to Si atoms whose close neighbors were strictly other Si atoms (Si(0Al) or Q4). The second, at −105 ppm, was due to Si(1Al) or Q3 sites and may also be related to acid sites [35], and, finally, the third signal at −99 ppm was consistent with Si(2Al) interaction or Q2.

The same occurred for the ^27^Al NMR spectra (Figure 3b), which only showed a peak at 55 ppm that was assigned to the structural aluminum, indicating that the difference in the particle size and crystallites did not cause any profound changes in the crystal structure of the samples.

The Si/Al molar ratios were calculated using ^29^Si NMR and ICP-OES chemical analysis. From the NMR results, the calculated ratios were 6.5 and 6.6 for the 170 °C and 150 °C samples, respectively, whereas the values estimated from the ICP analysis were 6.1 and 6.4 for the same samples, respectively. The results did not diverge significantly from each other when considering both measurements obtained by ICP and NMR, implying no significant differences in the overall elemental composition or within the zeolite networks. Although both samples presented considerably similar values, the mordenite obtained at 170 °C showed the highest total amount of aluminum in its structure.

Additionally, the mother liquor of the samples prepared for 8 h–8 h 30 min h at 170 °C was collected before the thermal treatment and analyzed by ICP to verify the silicon concentration in the solution. The obtained data were 256.2, 2256.0, and 3488.0 ppm for samples treated for 8 h, 8 h 15, and 8 h 30, respectively, suggesting that silicon flowed from the solid phase to the solution during the crystal growth step.

### 3.3. Influence of Gel Dilution

**Figure 4:**
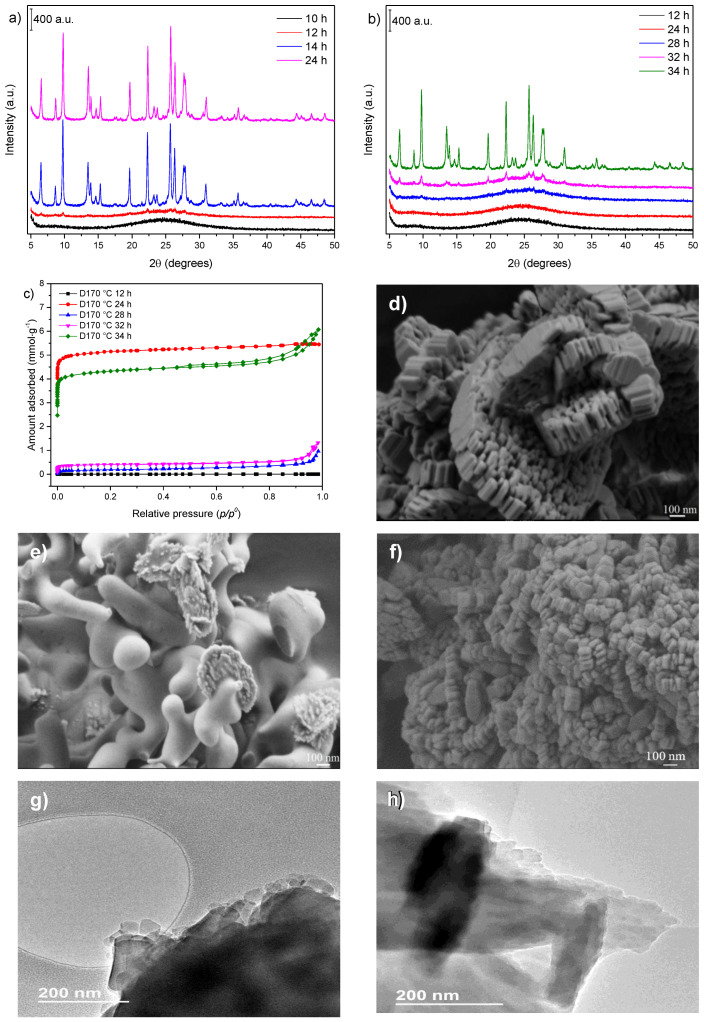
Comparison of crystallization kinetics for diluted gels. XRD patterns of samples obtained at (**a**) 170 °C and (**b**) 150 °C; (**c**) N_2_ adsorption–desorption isotherms; SEM images of samples prepared at (**d**) 170 °C for 24 h, (**e**) 150 °C for 28 h, and (**f**) 150 °C for 34 h; TEM images of samples synthesized at (**g**) 170 °C for 24 h and (**h**) 150 °C for 34 h.

**Figure A3:**
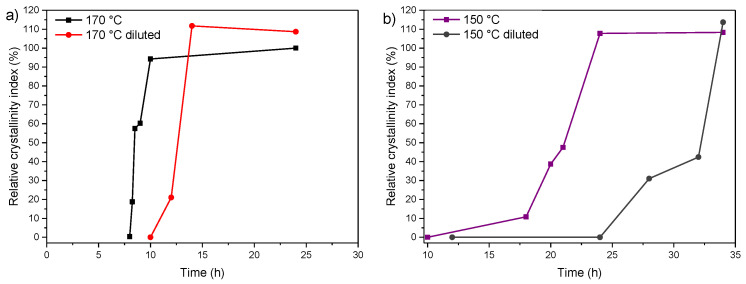
Comparative crystal growth curves for diluted and nondiluted sample series at 170 °C (**a**) and 150 °C (**b**).

The effect of the as-prepared gel dilution at 50 wt% on zeolite crystallization could be accompanied by X-ray diffraction (Figure 4a,b). At both temperatures (170 and 150 °C), the dilution caused a reduction in the rate of crystallization. This delay in the crystal growth rate when the gel was diluted could be understood as an increase in the dispersion of the precursor components of crystals, requiring more time for the attachment of species and particles involved in the crystallization. A comparison between the crystallization curves for the undiluted and diluted samples at 170 °C (Figure A3a) illustrates the difference in the crystallization rate.

The synthesis kinetics of the diluted gel used to synthesize the samples at 150 °C presented a similar behavior to that observed for the synthesis performed at 170 °C, as seen from the crystallization curves (Figure A3b). Although the growth rate decreased in both cases, the sample with the higher relative crystallinity index formed more rapidly than the cases of undiluted gels.

Regarding the N_2_ adsorption–desorption isotherms (Figure 4c), for the samples prepared at 170 °C (Entries 11–12), the zeolitic phase exhibited only micropores, and its ultimate textural properties were similar to those of the nondiluted sample (Entry 4). However, an increase in the N_2_ adsorption capacity at higher relative pressures (Figure 4c) was observed for the samples obtained at 150 °C (Entries 8–10). Concurrently, the hysteresis indicated some interparticle mesoporosity that was not seen in the nondiluted sample (Entry 7). Although the sample prepared at 150 °C for 34 h (Entry 10) showed a smaller specific surface area when compared to the sample at 150 °C for 24 h for the nondiluted gel (Entry 7), the former showed a higher external surface area, which could indicate an incomplete phase transition, pointing to higher control of zeolite properties under these synthesis conditions.

**Figure A4:**
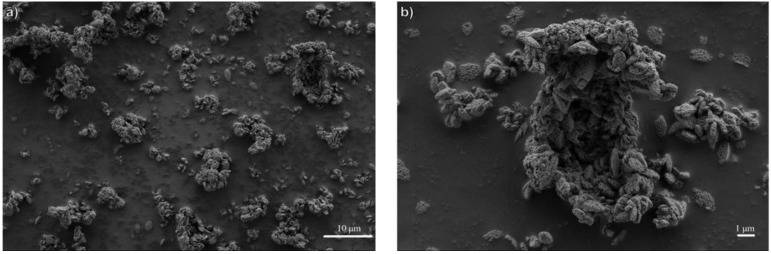
SEM images showing that the agglomerate of the diluted gel sample obtained at 170 °C do not aggregate in the spherical shape found in other samples (**a**) and the particles present smaller aggregation compared to the nondiluted samples (**b**).

The SEM analysis of the sample prepared after 24 h of heat treatment at 170 °C (Figure 4d) showed that the zeolite crystals were well-formed. The morphology of the particles presented similarities to that of the sample obtained under the same conditions using an undiluted gel (Figure 2d). The differences between them concerned the size of the crystallites, which, for diluted samples, reached 150–50 nm, and some particles presented a reduced aggregation between these crystallites. This may also be the reason for the hysteresis in the N_2_ isotherm of the sample of 34 h at 150 °C. The dilution seemed to hinder the formation of the clusters found in the nondiluted samples (Figure A4a,b).

Several studies have examined the influence of water on zeolite synthesis. Ding and Zheng [36] synthesized a β zeolite and noticed that high water content also promoted some delay in the crystallization rate. However, samples with higher crystallinity were obtained faster when using less water. Yakimov et al. [37] reported that using a more aqueous synthesis gel decreased the crystallization rate, but the size of the crystals increased.

Although the species are more dispersed in diluted systems, dilution also promotes greater mobility due to the reduced viscosity of the reaction medium. On the other hand, higher supersaturations promote reactions that give rise to zeolite crystals. Therefore, it is necessary to find a working range.

The SEM image of the sample prepared after 28 h of crystallization (Figure 4e) indicated that the mordenite phase appeared to grow on the surface of the amorphous phase. The particles of the formed phase resembled aggregated flakes. The SEM image of the sample synthesized during 34 h of thermal treatment (Figure 4f) showed particles with a well-defined morphology, with a behavior similar to that of the sample prepared at 170 °C. Moreover, the crystallites displayed nanosizes in the 125–35 nm range.

Further details regarding the crystallites can be extracted from the TEM images. The image of the sample synthesized at 170 °C for 24 h (Figure 4g) showed that, in general, the particles had well-distinguishable nanocrystallites on their surface. With crystallite sizes of less than 200 nm on average, fewer aggregates can be seen in Figure 4g, and they tended to be elongated in one direction, similar to those found by Kurniawan et al. [38], but slightly smaller. The TEM image of the sample synthesized at 150 °C for 34 h (Figure 4h) showed that the finned aspect was preserved.

The electron microscopy images aided in visualizing when the system was in the midst of the crystallization process and showed that the system could be interpreted according to recent zeolitization mechanisms [39,40]. Therefore, some particles with zeolite morphologies (the sample at 28 h) were dispersed in a different material, most likely amorphous, and a reorganization occurred to some extent in this solid phase to feed the nascent zeolite particles and generate new crystal growth sites.

**Figure 5:**
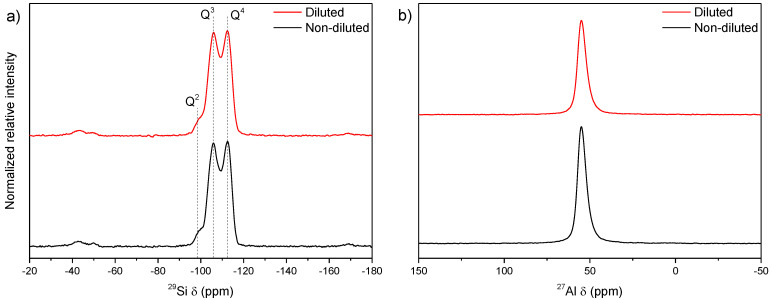
Solid-state NMR spectra of (**a**) ^29^Si and (**b**) ^27^Al in the samples synthesized for 24 h at 170 °C using nondiluted and diluted gels.

The solid-state NMR spectra of the diluted and nondiluted samples (Figure 5) were similar to the NMR spectra of the samples synthesized at 170 °C and 150 °C for 24 h (Figure 3), showing that dilution did not interfere with the silicon/aluminum ratio. The Si/Al molar ratio calculated by NMR was 6.5 for the nondiluted samples and 6.4 for the diluted samples, whereas the ratios derived from the ICP chemical analysis were 6.1 and 6.7, respectively.

As previously observed in the section on temperature variation, there were no appreciable differences between the samples, suggesting that the changes in crystallite size did not cause variations in the framework chemical composition.

### 3.4. Influence of the Sodium Content

**Figure 6:**
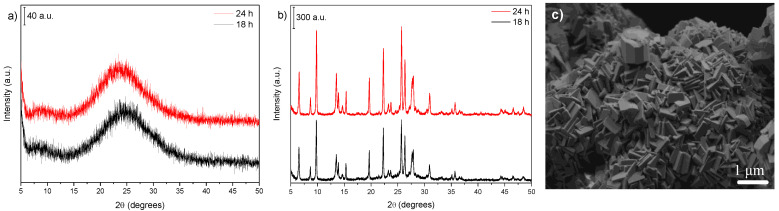
XRD patterns of samples synthesized using (**a**) Si/Na molar ratio of 7.5 and (**b**) Si/Na molar ratio of 2.5, and (**c**) SEM image of the sample prepared for 24 h using Si/Na = 2.5.

Since sodium is an essential component for the formation of MOR topology, a synthesis procedure was carried out using two other sodium contents in the gel compared to the reference of this work (Si/Na molar ratio = 3.5): Si/Na = 2.5 (the same as the IZA recipe) and Si/Na = 7.5. The XRD patterns (Figure 6a,b) showed that the sodium concentration significantly affected the crystal growth rate. Thus, when using the higher Si/Na ratio (i.e., a lower Na concentration), mordenite was not formed, whereas the crystallization was faster when a higher Na content was applied.

A significant increase in the particle size with the increase in the sodium content was observed for the SEM image, as shown in Figure 6c. For this sample (prepared for 24 h using Si/Na = 2.5), the sizes were in the 600–200 nm range, and the typical morphology of the mordenite phase was displayed. Thus, a gel with higher alkali concentrations is required to produce finned particles.

**Figure 7:**
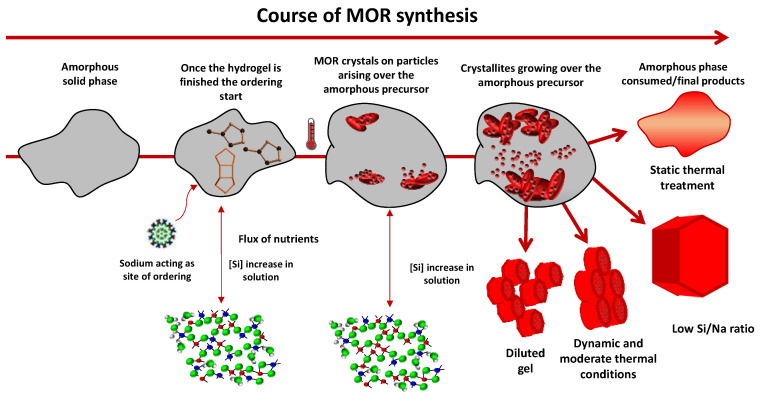
Schematic representation of mordenite particle formation based on the results of this study.

The results presented in this work can be interpreted based on some proposed pathways for the formation of zeolites [39,40]. A proposed scheme for the formation of mordenite particles is shown in Figure 7. From this perspective, crystallization could occur from the organization of silicon species on the surfaces of amorphous particles.

As previously shown according to the silicon analysis of the mother liquor, there was a constant flux of the silicon species originating from the dissolution of the amorphous particles. These silicon species were transported via the solution, being organized on the surface of the same amorphous phase in the form of small crystallites, subsequently giving rise to larger aggregated particles.

Nevertheless, the SEM and TEM images still showed the presence of crystals and aggregates growing on the surface of some amorphous material. This process occurs until the complete dissolution of the amorphous material has occurred.

## 4. Conclusions

Finned particles of mordenite zeolite were synthesized by controlling common synthesis parameters. Synthesis under agitation conditions increased crystal growth without harming the formation of nanocrystallites. As expected, the temperature was found to be a crucial factor in size control but was not completely determinant. The dilution of the gel was also shown to exert a significant effect on the crystals, making the growth process less sudden and slightly reducing the crystallite aggregation. Moreover, taking into account the IZA synthesis, the sodium content could be reduced, allowing a reduction in the crystal growth rate and a consequent decrease in crystallite size. Additionally, the results presented in this work showed how the formation of mordenite zeolite occurred from a dense gel. From the beginning of the heat treatment, the entities that gave rise to crystals were already formed and underwent a transition to an amorphous precursor during the induction period, continuing through the stage of crystal growth until all the constituent materials were consumed. The crystals aggregated, becoming crystallites that made up larger particles, which grew during the amorphous phase in a way that depended, above all, on the effects of variations in temperature and the heat treatment conditions.

## Figures and Tables

**Table 1:** Textural properties of synthesized samples.

Entry	T (°C)	Time	Condition	*S*_BET_ (M2/G)	*V*_μP_ (CM3/G)	*V*_TP_ (CM3/G)	*S*_EXT_ (M2/G)
**1**	170	8 h 15 ^a^	32 rpm	70	0.02	0.04	12
**2**	170	8 h 30 ^a^	32 rpm	170	0.05	0.08	26
**3**	170	10 h	32 rpm	240	0.08	0.10	28
**4**	170	24 h	32 rpm	470	0.16	0.17	50
**5**	170	10 h	Static	3	0	0	3
**6**	150	20 h	32 rpm	50	0.02	0.02	6
**7**	150	24 h	32 rpm	470	0.15	0.23	70
**8**	150	28 h	32 rpm D ^b^	15	0	0.03	12
**9**	150	32 h	32 rpm D	34	0.01	0.04	14
**10**	150	34 h	32 rpm D	390	0.12	0.21	66
**11**	170	12 h	32 rpm D	11	0	0.02	9
**12**	170	24 h	32 rpm D	470	0.15	0.19	58

^a^ 15 and 30 stand for 15 and 30 min, respectively. ^b^ D refers to diluted gel.

## Data Availability

Not applicable.
